# Selective deletion of interleukin-1 alpha in microglia does not modify acute outcome but may regulate neurorepair processes after experimental ischemic stroke

**DOI:** 10.1177/0271678X251323371

**Published:** 2025-03-20

**Authors:** Eloïse Lemarchand, Alba Grayston, Raymond Wong, Miyako Rogers, Blake Ouvrier, Benjamin Llewellyn, Freddie Webb, Nikolett Lénárt, Ádám Dénes, David Brough, Stuart M Allan, Gregory J Bix, Emmanuel Pinteaux

**Affiliations:** 1 Division of Neuroscience, School of Biological Sciences, Faculty of Biology, Medicine and Health (FBMH), The University of Manchester, Manchester, UK; 2 Geoffrey Jefferson Brain Research Centre, University of Manchester, Northern Care Alliance NHS Foundation Trust, The Manchester Academic Health Science Centre, Manchester, UK; 3 Department of Neurosurgery, Clinical Neuroscience Research Center, Tulane University School of Medicine, Orleans, LA, USA; 4 “Momentum” Laboratory of Neuroimmunology, HUN-REN Institute of Experimental Medicine, Budapest, Hungary

**Keywords:** Conditional gene knockout, interleukin-1 alpha, microglia, neurorepair, stroke

## Abstract

Inflammation is a key contributor to stroke pathogenesis and exacerbates brain damage leading to poor outcome. Interleukin-1 (IL-1) is an important regulator of post-stroke inflammation, and blocking its actions is beneficial in pre-clinical stroke models and safe in the clinical setting. However, the distinct roles of the two major IL-1 receptor type 1 agonists, IL-1α and IL-1β, and the specific role of IL-1α in ischemic stroke remain largely unknown. Here we show that IL-1α and IL-1β have different spatio-temporal expression profiles in the brain after experimental stroke, with early microglial IL-1α expression (4 h) and delayed IL-1β expression in infiltrated neutrophils and a small microglial subset (24–72 h). We examined for the first time the specific role of microglial-derived IL-1α in experimental permanent and transient ischemic stroke through microglial-specific tamoxifen-inducible Cre-loxP-mediated recombination. Microglial IL-1α deletion did not influence acute outcome after ischemic stroke. However, microglial IL-1α knock out (KO) mice showed reduced peri-infarct vessel density and reactive astrogliosis at 14 days post-stroke, alongside long-term impaired functional recovery. Our study identifies for the first time a critical role for microglial IL-1α on post-stroke neurorepair and recovery, highlighting the importance of targeting specific IL-1 mechanisms in brain injury to develop effective therapies.

## Introduction

Inflammation is a major contributor to stroke pathogenesis, and the role of inflammation driven by the pro-inflammatory cytokine interleukin-1 (IL-1) during post-stroke injury has been the focus of intense research. Indeed, pre-clinical studies have demonstrated the deleterious actions of IL-1 after stroke, whilst blocking its actions with the IL-1 receptor antagonist (IL-1Ra) is beneficial in experimental stroke^1^ and in clinical setting.^2^ The cellular targets and actions of IL-1 in stroke are unclear, although IL-1 receptors (primarily the IL-1 type 1 receptor, IL-1R1) are expressed mainly on brain endothelial cells, and we have recently demonstrated, using cell-specific conditional gene deletion of IL-1R1, that brain endothelial cells and neurons are key targets of IL-1 in stroke.^3^ IL-1α and IL-1β are the two main agonists of the 11 members in the IL-1 family, and they are expressed centrally and peripherally after stroke.^4,5^ While the role of IL-1β has received more attention in the context of ischemic stroke, the precise role and mechanisms of action of IL-1α remain largely unknown. However, early studies suggested that IL-1α and IL-1β may exert specific differential actions during inflammation^6,7^ and, more recently, these isoforms were found to show a differential expression after experimental stroke, with early IL-1α expression in microglia preceding that of IL-1β.^8^ Furthermore, our recently published work found that IL-1α, but not IL-1β, selectively triggers angiogenesis *in vitro*^9^ and IL-1α given at a low dose triggers angiogenesis and improves functional recovery after stroke,^10^ suggesting a specific role for IL-1α distinct to that of IL-1β after stroke.

Here we show that brain-derived IL-1α is expressed exclusively by microglia early after stroke, followed by a later IL-1β expression in infiltrated neutrophils and a small microglial subset. Using a genetic mouse model allowing cell-specific deletion of IL-1α, we examined the specific contribution of microglial-derived IL-1α in ischemic stroke. Selective microglial IL-1α deletion did not modify acute outcome but led to impaired neurorepair processes and long-term functional recovery after experimental ischemic stroke. These data reveal a potential critical role for microglial IL-1α in stroke and highlight the need for a much greater understanding of the respective contributions of IL-1 family members to cerebral ischemia, to allow the most efficient treatments to be developed.

## Materials and methods

### Animals

All animal procedures were carried out in accordance with the Animals (Scientific Procedures) Act (1986), under a Home Office UK project license, approved by the local Animal Welfare Ethical Review Board (University of Manchester), and experiments were performed in accordance with ARRIVE (Animal Research: Reporting of In Vivo Experiments) guidelines,^11^ with researchers blinded to genotype.

Animals were housed at 21 ± 1°C, 55 ± 10% humidity on a 12 h light-dark cycle in Sealsafe Plus Mouse individually ventilated cages (Techniplast, Italy). Mice were supplied with Sizzle Nest nesting material (Datesand Ltd., UK) and cardboard enrichment tubes (Datesand Ltd., UK), had *ad libitum* access to standard rodent diet (SDS, UK) and water, and were acclimatized to the facility for at least 1 week before any experimental work.

**Figure 1.**
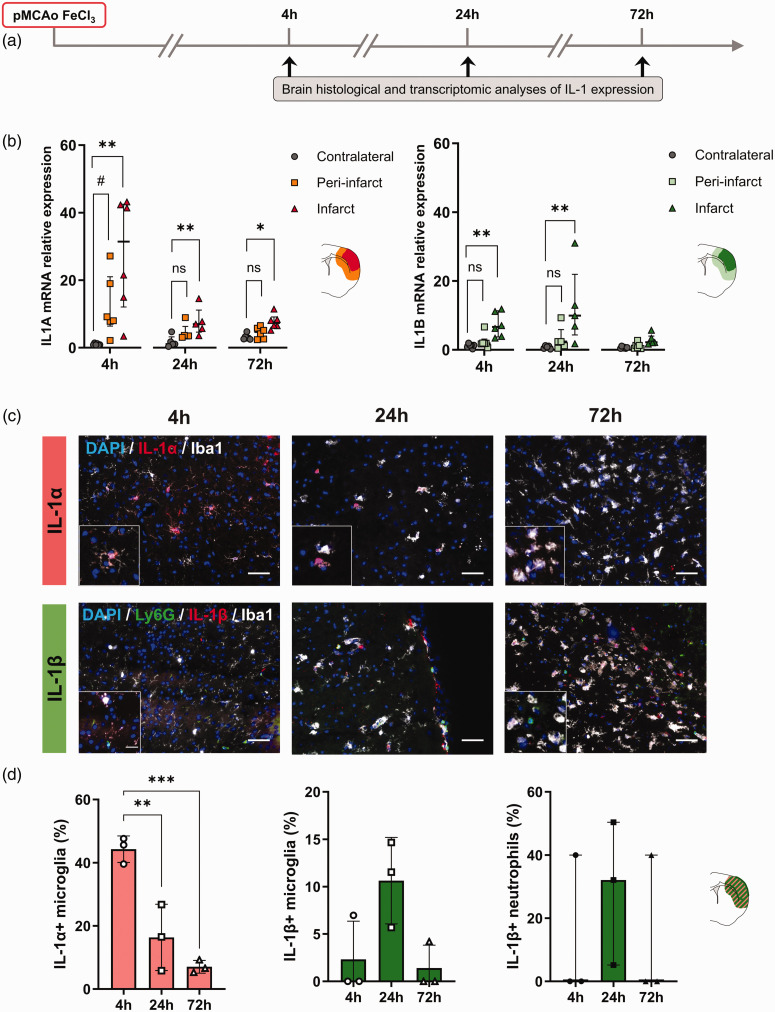
IL-1 brain expression during the early phase of distal pMCAo. (a) Schematic representation of the experimental design. (b) RT-qPCR analysis showing different spatio-temporal expression of *IL1A* (left) and *IL1B* (right) in the contralateral, peri-infarct and infarct areas at 4, 24 and 72 h after stroke (mRNA levels normalized to contralateral at 4 h); n = 6/group, ns = non-significant, ^#^p < 0.100, *p < 0.05, **p < 0.01 vs. contralateral, Friedman test followed by Dunn’s post hoc test. (c) Representative immunostaining of IL-1α (red), microglia (Iba1, white), neutrophils (Ly6G, green), IL-1β (red), and DAPI (blue) in the infarct at 4, 24 and 72 h after stroke. Scale bar is 50 µm (large image) and 20 µm (insert) and (d) Percentage of IL-1α+ microglia in the infarct at 4, 24 and 72 h after stroke. (left; n = 3/group, ***p < 0.001, one-way ANOVA followed by Dunnet’s post hoc test). Percentage of IL-1β+ microglia and IL-1β+ neutrophils in the contralateral, peri-infarct and infarct areas at 4, 24 and 72 h after stroke. (right; n = 3/group, one-way ANOVA and Kruskal-Wallis followed by Dunnet’s or Dunn’s post hoc test, respectively). All data are shown as median (IQR), except graphs in (d) showing the percentage of IL-1α+ and IL-1β+ microglia, where data are shown as mean ± SD.

**Figure 2.**
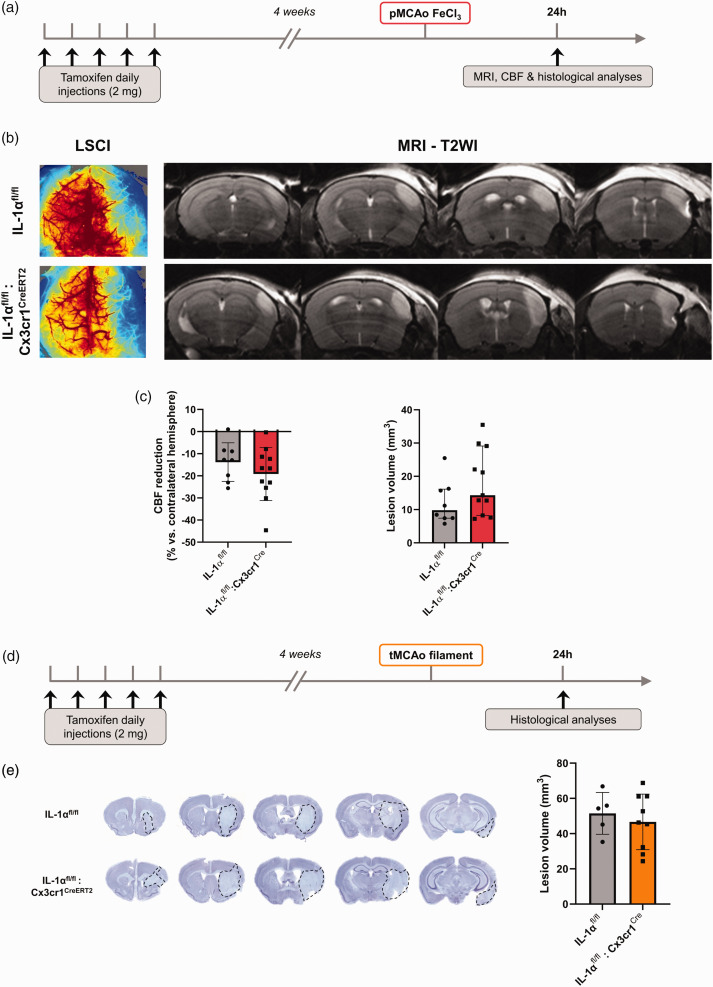
Microglial IL-1α deletion does not influence acute brain damage after pMCAo nor tMCAo. (a) Schematic representation of the experimental design to study acute outcome after pMCAo. (b) Representative LSCI (left) and T2-WI (right) and (c) CBF quantification (left) and lesion volume quantification (right) at 24 h after pMCAo in IL-1α^fl/fl^:Cx3cr1^CreERT2^ mice. (d) Schematic representation of the experimental design to study acute outcome after tMCAo and (e) representative cresyl violet-stained brains (lesion delineated by dashed lines) and lesion volume quantification (n = 5–9/group, unpaired t-test). All data are shown as mean ± SD, except the graph in (c) showing the lesion volume at 24 h post-pMCAo, where data are shown as median (IQR).

**Figure 3.**
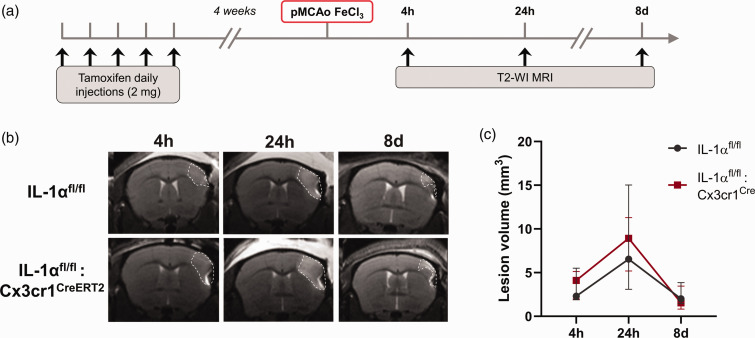
Microglial IL-1α deletion does not influence brain lesion progression up to 8 days after pMCAo. (a) Schematic representation of the experimental design. (b) Representative T2-WI at 4 h, 24 h and 8 days after pMCAo and (c) Lesion volume quantification showing no differences between IL-1α^fl/fl^ and IL-1α^fl/fl^:Cx3cr1^CreERT2^ mice (n = 4/group, mixed effects model (REML) followed by Sidak’s post hoc test). Data are shown as mean ± SD.

**Figure 4.**
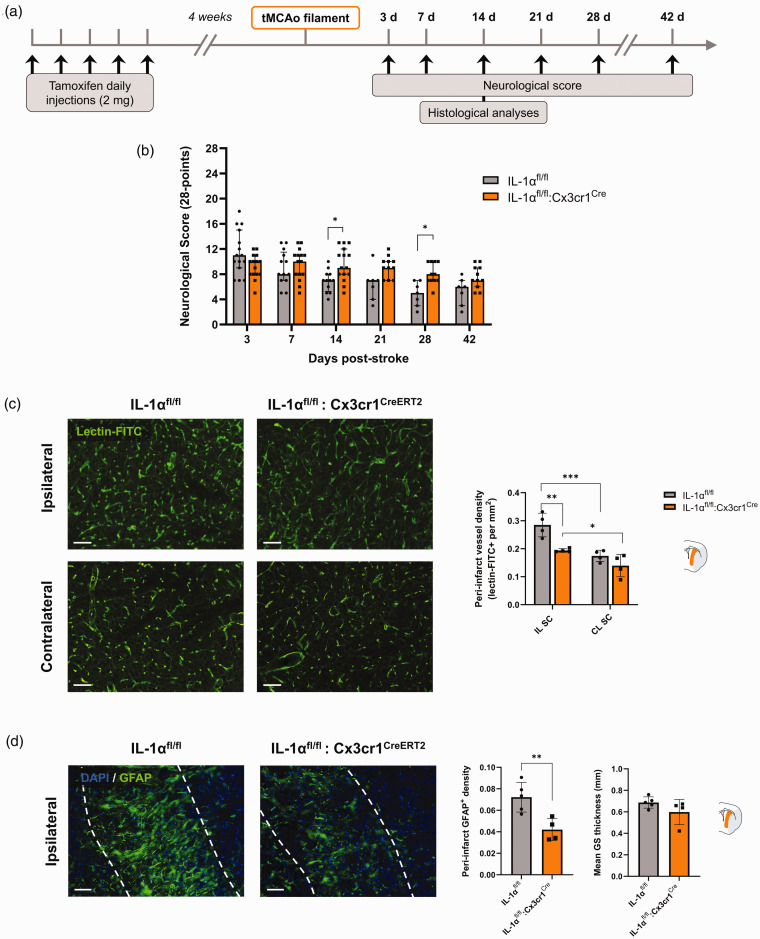
Effect of microglial IL-1α deletion on long-term stroke outcome. (a) Schematic representation of the experimental design. (b) 28-point neurological score showing significant functional deficit IL-1α^fl/fl^:Cx3cr1^CreERT2^ compared to in IL-1α^fl/fl^ mice, 14 and 28 days after tMCAo (n = 15–16, *p < 0.05 mixed effects model (REML) followed by Sidak’s post hoc test). (c) Representative immunostaining of vascular density (lectin-FITC+) in the ipsilateral peri-infarct and corresponding contralateral subcortical brain regions in IL-1α^fl/fl^ and IL-1α^fl/fl^:Cx3cr1^CreERT2^ mice, and quantification (Scale bar: 50 µm, n = 4/group, *p < 0.05, **p < 0.01, ***p < 0.001, two-way ANOVA followed by Sidak’s post hoc test) and (d) Representative immunostaining of the glial scar (GFAP+) in the ipsilateral peri-infarct subcortical brain region in IL-1α^fl/fl^ and IL-1α^fl/fl^:Cx3cr1^CreERT2^ mice, and quantification of the GFAP+ density (left) and glial scar (GS) thickness (right) (Scale bar: 50 µm, n = 4–5/group, unpaired t-test, **p < 0.01). Data are shown as mean ± SD.

Experiments were performed on a total of 21 C57BL/6NCrl male mice (Charles River, UK) and on a total of 104 C57BL/6J male mice from an in-house colony (IL-1α^fl/fl^:Cx3cr1^Cre^^ERT2^ and IL-1α^fl/fl^ as littermate controls) at the University of Manchester. Complete schemes of the experimental designs are presented in Figures 1 to 4 and Figures S1 to S7. A total of 18 mice were excluded: 11 due to experimental failure/complications, 1 due to mortality post-intervention and 6 culled due to declining post-stroke health issues. Microglial specific IL-1α KO mice were generated by crossing IL-1α^fl/fl^ mice in which exon 4 of the *IL1A* gene is flanked with loxP sites^12^ with Cx3Cr1^CreERT2^ mice expressing CX3CR1 promoter-driven Cre recombinase (JAX stock #020940)^13^ that is expressed in the mononuclear phagocyte lineage. To induce Cre^ERT2^ activity and subsequent Cre-loxP-mediated deletion of IL-1α in CX3CR1-expressing cells, 3–6 months old mice were given tamoxifen by intraperitoneal injection for 5 consecutive days (2 mg/100 µL in corn oil, 75 mg/kg, Sigma-Aldrich). Control mice were Cre^−/−^ IL-1α^fl/fl^ treated with tamoxifen. Mice recovered for 4 weeks after tamoxifen treatment to allow the peripheral myeloid wild type (WT) population to replenish, with sustained IL-1α deletion in microglia.^14^ Ischemic stroke was induced at 4–7 months of age, regardless of tamoxifen treatment.

### Focal cerebral ischemia

Two different surgical models were used to induce a permanent or transient middle cerebral artery occlusion (pMCAo and tMCAo, respectively), both as clinically relevant models of focal ischemic stroke, the former recapitulating ischemic stroke with platelet-rich thrombi and unsuccessful reperfusion, and the latter mimicking large vessel occlusions followed by recanalization by endovascular thrombectomy.

Distal FeCl_3_-induced pMCAo was performed as a model of thrombotic cerebral ischemia, as described previously.^15^ In brief, mice were anesthetized with isoflurane (4% for induction and 2% for maintenance in 30:70% mixture of N_2_O/O_2_) and placed in a stereotaxic device. A small craniotomy (1 mm diameter) was performed on the parietal bone to expose the right MCA. A Whatman filter paper strip soaked in FeCl_3_ (40%, Sigma-Aldrich) was placed on the dura mater on top of the MCA for twice 5 min. Cerebral blood flow (CBF) in the MCA territory was measured continuously by laser Doppler flowmetry (Oxford Optronix, UK). A total of 11 mice were excluded due to clot instability leading to inadequate occlusion and absence of ischemic lesion.

Transient focal cerebral ischemia was induced in two different research centers using the intraluminal model of proximal tMCAo, as previously described.^3^ Briefly, mice were anesthetized with isoflurane (4% for induction and 2% for maintenance in 30:70% mixture of N_2_O/O_2_, a midline incision was made on the ventral surface of the neck and the left common carotid artery isolated and ligated. A 6-0 monofilament (Doccol, Sharon, MA, USA) was introduced into the internal carotid artery via an incision in the common carotid artery (30 min MCAo) or in the external carotid artery (45 min MCAo). The filament was advanced approximately 10 mm into the common carotid with the filament making its way distal to the carotid bifurcation, to occlude the MCA. A 10 mm mark was made on the filament to visualize the required length to be inserted beyond the carotid bifurcation. There was an attrition of animals in the experimental groups followed up to 42 days after tMCAo, with 1 spontaneous death within the first 24 h, 4 animals culled on day 1, 1 culled at day 3 and 1 culled at day 4 relating to declining post-stroke health issues.

Body temperature was monitored and maintained at 37 ± 0.5°C throughout both procedures using a homoeothermic blanket and rectal probe (Harvard Apparatus, UK). Topical anesthetic (EMLA, 5% prilocaine and lidocaine, AstraZeneca, UK) was applied to skin incision sites prior to incision. Buprenorphine (0.05 mg/kg) was administered subcutaneously at the time of surgery and at 24 h. Mice were weighed daily for the first week post-stroke, given mashed diet and administered with 0.5 mL saline daily until body weight stabilized (approximately at 4 days post-stroke).

### Laser speckle contrast imaging (LSCI)

At 24 h after distal pMCAo, a laser speckle contrast imager (moorFLPI-2, Moor Instruments, UK) was used to measure cortical CBF. Briefly, mice were anesthetized with isoflurane (4% for induction and 2% for maintenance in 30:70% mixture of N_2_O/O_2_) and fixed in a stereotaxic frame. The scalp was exposed by a midline skin cut, and the skin was secured away using surgical clips. An ultrasound gel was applied to the mouse skull and a glass coverslip was mounted to improve image quality. The CBF was recorded by LSCI for 3 min (20 ms exposure time, 25 frame filter). Regions of interest (ROI) were drawn on the ipsilateral and contralateral brain hemispheres and analyzed using the moorFLPI Review V4 software (Moor Instruments). The percentage of CBF loss was calculated in the ipsilateral hemisphere compared to the contralateral hemisphere.

### Magnetic resonance imaging (MRI)

Anesthetized mice underwent T2-weighted MRI scans on Bruker Advance III console (Bruker Biospin Ltd, UK) using a 7 Tesla magnet at 4 h, 24 h and 8 d post-pMCAo. T2-weighted images were acquired using a multi-slice multi-echo (MSME) sequence: TE/TR 35/7320 ms with 125 × 125 × 500 µm^3^ spatial resolution. T2-weighted images were used to quantify lesion volume using ImageJ. The area of the T2 hypersignal (corresponding to the brain lesion/edema) was measured using ImageJ software and lesion volume was calculated by multiplying the areas by the axial slice thickness (0.5 mm).

### Neurological deficit scoring

Mice were scored for neurological focal deficits using a 28-point neurological scoring system previously described.^16^ This is a cumulative score based on the following categories: body symmetry, gait, climbing, circling, front limb symmetry, compulsory circling, and whisker response. Animals are ranked from 0 (normal) to 4 (extreme deficit) for each category. Neurological score was performed at 3, 7, 14, 21, 28 and 42 d post-stroke in the intraluminal tMCAo model, which allows for a better assessment of behavioral deficits beyond the acute phase post-stroke, as opposed to distal cortical stroke models, which limit the investigation of long-term functional outcome.

### Tissue processing

Anesthetized mice were transcardially perfused with cold saline followed by 50 mL of 4% paraformaldehyde (PBS 0.1 M, pH 7.4). Brains were removed, post-fixed in 4% paraformaldehyde and cryoprotected in sucrose 30% for 24 h, and snap-frozen in isopentane at −30°C. Brains were then cut to a thickness of 30 μm using a freezing sledge microtome (Bright Instruments, UK) and then stored in cryoprotectant (0.05 M Na_2_HPO_4_*2H_2_O, 5 mM NaH_2_PO_4_, 30% anhydrous ethylene glycol, 20% glycerol) at −20°C until required for free-floating immunohistochemistry, or embedded in optimal cutting temperature compound (OCT, PFM Medical UK, Ltd) prior to snap-freezing and cutting to a thickness of 10 μm using a cryostat (Leica CM1950, UK) for on-slide immunohistochemistry.

### Cresyl violet stain

Cresyl violet staining was performed to measure lesion volume after tMCAo, as previously described.^17^ Briefly, 30 μm-thick brain sections were stained with 1% cresyl violet, and cover-slipped with DPX mounting medium (06522, Sigma-Aldrich). For each brain, infarct volumes were measured using image J on deﬁned coronal sections across the whole brain (14-16 coronal sections per animal), spaced at approximately 360 µm apart. Each coronal section, with its brain co-ordinates relative to Bregma and lesion was integrated to estimate total lesion volume for each brain and corrected for edema. Lesion volume was determined as the area under the curve, by plotting the measured area for each coronal brain slice (Y-axis) and their respective coordinates from Bregma (X-axis).

### Immunohistochemistry

Brain sections were washed in Dulbecco’s phosphate buffeted saline (DPBS) with 0.1% Tween20, incubated in heat-mediated antigen retrieval in Tris-EDTA pH 8.6 solution for 20 min in a water bath set to 95°C, and incubated in blocking solution (1% BSA and 5% donkey serum in DPBS with 0.05% Tween20, 0.1% Triton X-100 and 0.2 M glycine) for 1 h at room temperature. Sections were then incubated with rat–anti-Lymphocyte antigen 6 complex locus G6D (Ly6G) (1.25 μg/mL on-slide, 0.67 μg/mL free-floating, 127602; Biolegend), anti-Ionized calcium-binding adaptor molecule 1 (Iba1) (2 µg/mL on-slide, 1 µg/mL free-floating, ab178846, Abcam), goat–anti-IL-1α (1 μg/mL, AF-400-NA; R&D Systems), goat–anti-IL-1β (1 μg/mL, AF-401-NA; R&D Systems), goat–anti-Intercellular Adhesion Molecule 1 (ICAM-1) (1 μg/mL, AF796; R&D Systems), and chicken-anti-glial fibrillary acidic protein (GFAP) (1 μg/mL, Antibodies, ab4674) antibodies overnight at 4°C (in 1% BSA and 0.3% Triton X-100 in PBS). The following secondary antibodies were incubated for 2 h at room temperature: Alexa Fluor 488 donkey anti-rat, goat anti-chicken and donkey anti-goat (10 µg/mL; A21208, A11039, A32814), Alexa Fluor 568 donkey anti-rabbit (10 µg/mL; A10042), and Alexa Fluor 647 donkey anti-rat, donkey anti-rabbit (10 µg/mL; A48272, A31573). IL-1β and IL-1α signal was amplified with Tyramide SuperBoostTM (Thermo Fisher) using biotinylated horse–anti-goat IgG (7.5 µg/mL, BA-9500; Vector) secondary antibody. To assess vessel density, brain slices were stained with DyLight-488 conjugated tomato lectin (1:200, ThermoFisher, L32470) during secondary antibody incubation. Sections were counterstained with DAPI (25 ng/mL in H_2_O) to stain for cell nuclei, and mounted with Prolong Gold antifade reagent (Thermo Fisher Scientific; P36934).

Images were collected on Zeiss Axioimager.M2 upright microscope using a 20X Plan Apochromat objective and captured using a Coolsnap HQ2 camera (Photometrics) through Micromanager software (v1.4.23), or on a 3 D-Histech Pannoramic-250 microscope slide-scanner using a x20/0.30 Plan Achromat objective (Zeiss, Germany) and captured using the Case Viewer software (3 D-Histech, Hungary). All quantitative analyses were performed on at least 2 regions of interest (ROIs) per defined brain area (peri-infarct or infarct cortical/subcortical areas, and equivalent contralateral cortical/subcortical areas), in 3 different coronal brain slices per animal, at equivalent coordinates relative to Bregma. All images were processed using ImageJ, except for microglial activation based on morphology (Iba-1+ cells), neutrophil infiltration (Ly6G+ cells), and vessel activation (ICAM-1+), which were analysed using a pixel and subsequent object classification using the Ilastik software program.^18^ To train the pixel classification algorithm, labelling was performed on at least 6 randomly selected images for each immunofluorescence marker. All color/intensity, edge, and texture features were included, with a σ0 = 0.30, σ1 = 0.70, σ2 = 1.00, σ3 = 1.60, σ4 = 3.50, σ5 = 5.0 and σ6 = 10.0. Object classification was then applied to train the machine learning software to discern amoeboid from ramified microglia (Iba1+) as resting or activated cells, respectively. To analyse vessel density (lectin-FITC+), simple segmentation predictions were exported upon pixel classification on Ilastik and analysed in ImageJ. For analyses performed on Image J, the threshold was equally adjusted for all images, and the area limited to threshold of each image was measured. All image analyses were performed blinded to experimental groups.

### Real-Time quantitative polymerase chain reaction

Total RNAs were extracted from samples with TRIzol Reagent (Thermo Fisher) according to the manufacturer. RNA (1 µg) was converted to cDNA using Super Script III Reverse Transcriptase (Thermo Fisher). Real-Time Quantitative polymerase chain reaction (RT-qPCR) was performed using Power SYBR Green PCR Master Mix (Thermo Fisher) in 384-well format using a 7900HT Fast Real-Time PCR System (Applied Biosystems). Three microliters of 1:20 diluted cDNA was loaded with 200 mmol/L of primers in triplicate. Data were normalized to the expression of the housekeeping gene Hmbs. Specific primers were designed using Primer3Plus software (http://www.bioinformatics.nl/cgi-bin/primer3plus/primer3plus.cgi). Primers used were: Il1b Forward—AACCTGCTGGTGTGTGACGTTC, Il1b Reverse—CAGCACGAGGCTTTTTTGTTGT, Il1a Forward—TCTCAGATTCACAACTGTTCGTG, Il1a Reverse—AGAAAATGAGGTCGGTCTCACTA. Expression levels of genes of interest were computed as follows: relative mRNA expression = E^−(Ct  of gene of interest)^/E^−(Ct of housekeeping  gene)^, where Ct is the threshold cycle value and E is efficiency.

### Statistical analysis

Statistical analyses were performed using GraphPad Prism 8.0. All values are expressed as mean ± standard deviation (SD) or median (InterQuartile Range, IQR) according to the normal or non-normal distribution of the represented variable, respectively. The normality of continuous variables was assessed using the Shapiro-Wilk test (n < 30) or Kolmogorov-Smirnov test (n ≥ 30). Data were analyzed using unpaired t-test, one-way ANOVA (followed by Dunnet’s multiple comparisons post hoc test), two-way ANOVA (followed by Dunnet’s or Sidak’s multiple comparisons post hoc test), or by fitting a mixed effects model using Restricted Maximum Likelihood (REML), followed by Sidak’s multiple comparisons post hoc test. For non-normally distributed variables, Mann-Whitney test, Kruskal Wallis test (followed by Dunn’s multiple comparisons post hoc test), or Friedman’s test for repeated measures (followed by Dunn’s multiple comparisons post hoc test), were used. Extreme outliers were defined as data points falling below Q1 – 3*IQR or above Q1 – 3*IQR and removed from subsequent analyses. The significant level was set at p < 0.05.

## Results

### IL-1α and IL-1β are differentially expressed during the acute phase of ischemic stroke

Gene expression of *IL1A* and *IL1B* was analyzed by RT-qPCR in the infarct, peri-infarct and contralateral brain cortical areas dissected from mice subjected to distal pMCAo at 4, 24 or 72 h post-stroke (Figure 1(a)). The expression of *IL1A* was significantly different across the dissected brain regions at 4, 24 and 72 h post-MCAo (Friedman test, p = 0.0017, p = 0.0085 and p = 0.0289). Specifically, a significant increase of *IL1A* mRNA was observed in the infarct area and a trend in increase in the peri-infarct area at 4 h after stroke (28-fold and 12-fold increase, p = 0.0030 and p = 0.0866 vs. contralateral, respectively); Figure 1(b). The expression of *IL1A* mRNA remained elevated in the infarct area at 24 h and 72 h post-MCAo (8-fold and 8-fold increase, p = 0.0089 and p = 0.0187 vs. contralateral, respectively), but decreased to similar levels in the peri-infarct area at 24 h and 72 h post-MCAo (5-fold and 4-fold increase, p = 0.1156 and p = 0.7730 vs. contralateral, respectively). The expression of *IL1B* was also significantly different across the dissected brain regions at 4 h and 24 h (Friedman test, p = 0.0120, p = 0.0085 and p = 0.0289), and 72 h post-MCAo, although not reaching statistical significance (Friedman test, p = 0.0521). However, the increase of *IL1B* mRNA expression was greater at 24 h than 4 h post-stroke in the infarct areas compared to the contralateral hemispheres (12-fold increase, p = 0.0089, and 7-fold increase, p = 0.0078, respectively), returning to similar levels at 72 h (p > 0.05 vs. contralateral). In contrast, *IL1B* mRNA levels in the peri-infarct areas were similar to the contralateral hemisphere at all timepoints (p > 0.05 vs. contralateral); (Figure 1(b)).

To assess the protein expression profile of IL-1 after stroke and identify the cell types expressing IL-1α and IL-1β, we performed immunostaining for both cytokines on brain sections. IL-1α was detected exclusively in microglia in the ipsilateral brain, with all IL-1α+ cells also being positive for Iba1. This expression of microglial IL-1α+ was significantly different across timepoints (one-way ANOVA, p = 0.0011), being greater at 4 h compared to 24 and 72 h. Specifically, 44% of Iba1+ cells were IL-1α+ at 4 h, compared to 16% and 7% at 24 and 72 h post-MCAo, respectively (p = 0.0037 and p = 0.0008 vs. 24 and 72 h, respectively); (Figure 1(c) and (d)). IL-1β expression in microglia peaked at 24 h and was significantly different across timepoints (one-way ANOVA, p = 0.0452), however, IL-1β expression was observed only in a small subset of microglia, peaking at 24 h after stroke (11% at 24 h vs. 2% and 1% at 4 and 72 h post-MCAo respectively), yet not reaching statistical significance between time points (p = 0.0626 and p = 0.9372 at 24 h vs. 4 and 72 h post-MCAo, respectively). In contrast, IL-1β was mostly expressed by neutrophils (Ly6G+), peaking at 24 h post-MCAo (29% at 24 h vs. 13% at 4 and 72 h post-MCAo, respectively), again with no differences between timepoints (p = 0.4815 and p = 0.9999 at 24 h vs. 4 and 72 h post-MCAo, respectively); Figure 1(c) and (d). The contralateral hemisphere was also investigated but no positive cells for either IL-1α nor IL-1β were observed (*data not shown*).

### Successful microglial IL-1α deletion does not affect acute ischemic stroke outcome

Based on our previous results showing that microglia are the major source of brain IL-1α acutely after stroke, we targeted microglial-derived IL-1α to investigate its specific role after stroke. Successful deletion of IL-1α in microglia was confirmed by immunohistochemistry in IL-1α^fl/fl^:Cx3cr1^CreERT2^ mice subjected to distal pMCAo at both 4 h (Fig. S1) and 24 h (Fig. S3) after stroke, which showed no IL-1α+ staining as opposed to IL-1α^fl/fl^ mice, which did show IL-1α+Iba1+ staining.

First, we investigated the influence of microglial IL-1α depletion on acute stroke outcome, at 24 h after both distal pMCAo and proximal tMCAo (Figure 2). At 24 h after pMCAo, IL-1α^fl/fl^:Cx3cr1^CreERT2^ mice showed no changes, neither in lesion volume (18.2 ± 9.9 mm^3^ vs. 12.2 ± 6.4 mm^3^ in the IL-1α^fl/fl^ group, p = 0.2060; Figure 2(a) and (b)), nor in % of CBF loss in the ipsilateral vs. contralateral brain hemisphere (−19% vs. −14% in the IL-1α^fl/fl^ group, p = 0.2991; Figure 2(a) and (b)). In order to assess infarct evolution beyond 24 h after pMCAo, IL-1α^fl/fl^:Cx3cr1^CreERT2^ mice and control IL-1α^fl/fl^ mice were followed for 8 days and underwent MRI to track infarct size longitudinally at 4 h, 24 h and 8 d post-MCAo by T2-weighted imaging (Figure 3). The infarct size significantly changed with time (p < 0.0001), however once again there were no changes in lesion volume associated to genotype (p = 0.9361) nor an interaction between genotypes and lesion volume progression with time (p = 0.8985). We also investigated whether deletion of microglial IL-1α could affect acute immune cell responses, at 24 h after pMCAo. As expected, microglial density (Iba1+ cells) was significantly different between brain regions (two-way ANOVA, p < 0.0001), with an increase in microglial cell density in the infarct areas in both IL-1α^fl/fl^:Cx3cr1^CreERT2^ (p = 0.0156 vs. contralateral, p = 0.0003 vs. peri-infarct), and control IL-1α^fl/fl^ mice (p = 0.0017 vs. contralateral, p = 0.0016 vs. peri-infarct). An increase in neutrophil infiltration was also observed in the infarct areas in both IL-1α^fl/fl^:Cx3cr1^CreERT2^ (p = 0.0357 vs. contralateral, p = 0.1099 vs. peri-infarct), and control IL-1α^fl/fl^ mice (p = 0.0098 vs. contralateral, p < 0.001 vs. peri-infarct). However, there were no changes between genotypes in neither microglia density nor neutrophil infiltration in the contralateral, peri-infarct and infarct areas (p > 0.05 for all comparisons vs. IL-1α^fl/fl^); (Fig. S2). Moreover, IL-1β expression in both microglia and neutrophils remained unchanged between genotypes (p > 0.05 for all comparisons vs. IL-1α^fl/fl^; Fig. S2). The percentage of IL-1β+ microglia was different across brain regions (p = 0.0068), showing a significant increase in IL-1β+ microglia in the infarct areas in both IL-1α^fl/fl^:Cx3cr1^CreERT2^ (p = 0.0442 vs. contralateral), and control IL-1α^fl/fl^ mice (p = 0.0207 vs. contralateral), yet no significant interaction observed between the expression across brain regions and genotype (p > 0.05 for all comparisons). The percentage of L-1β+ neutrophils was also different across brain regions (p = 0.0463), with no significant interaction between the expression across brain regions and genotype, nor significant differences between contralateral, peri-infarct and infarct areas in neither of the genotypes (p > 0.05 for all comparisons).

At 24 h after tMCAo, IL-1α^fl/fl^:Cx3cr1^CreERT2^ mice showed no changes in lesion volume (46.7 ± 15.8 mm^3^ vs. 51.5 ± 11.9 mm^3^ in the IL-1α^fl/fl^ group, p = 0.5662; Figure 2(d)). The lack of effect of microglial IL-1α on brain damage was reproduced in a different tMCAo model (45 min occlusion via an incision in the external carotid artery; p > 0.05 for all comparisons vs. IL-1α^fl/fl^) (Fig. S4). We also investigated whether the deletion of microglial IL-1α could affect acute immune cell responses, at 24 h post-tMCAo. Again, microglial density (Iba1+ cells) was significantly different between brain regions (two-way ANOVA, p = 0.0058), yet no significant differences were observed between specific brain regions in either of the genotypes (p > 0.05 for all comparisons). As expected, microglial activation, determined as the percentage of activated/amoeboid microglia (Iba1+ cells) was significantly different between brain regions (two-way ANOVA, p < 0.0001), with an increase in activated microglia in the infarct areas in both IL-1α^fl/fl^:Cx3cr1^CreERT2^ (p < 0.0001 vs. contralateral, p = 0.0002 vs. peri-infarct), and control IL-1α^fl/fl^ mice (p < 0.0001 vs. contralateral, p = 0.0015 vs. peri-infarct). Importantly, there were no differences in terms of microglial density or activation in the contralateral, peri-infarct and infarct areas between genotypes (p > 0.05 for all comparisons vs. IL-1α^fl/fl^). Endothelial activation, measured by ICAM-1 expression, and neutrophil infiltration were also similar in the ipsilateral brain in both genotypes (p > 0.05 for all comparisons vs. IL-1α^fl/fl^); (Fig. S5).

### Effect of microglial IL-1α expression on long-term outcome after ischemia-reperfusion model

We observed that animals with microglial IL-1α deletion had a worse neurological score at 14 d (9.6 ± 2.6 vs. 6.9 ± 1.7 in the IL-1α^fl/fl^ control group, p = 0.0228), at 21 d although not reaching statistical significance (8.1 ± 1.7 vs. 4.9 ± 2.0 in the IL-1α^fl/fl^ control group, p = 0.1792), and up to 28 d after tMCAo (9.2 ± 1.7 vs. 6.4 ± 2.6 in the IL-1α^fl/fl^ control group, p = 0.0229); (Figure 4(b)). Specifically, a mixed effects model (REML) showed an effect of time (p < 0.0001), but not genotype (p = 0.1756), although there was an interaction between time and genotype (p < 0.0001). The interaction effect indicates that the neurological score changed differently over time between genotypes, leading to significant differences at certain timepoints but not throughout the entire time course, which was then reflected by Sidak’s multiple comparisons post-hoc tests in a significant difference between genotypes at 14 days and 28 days post-stroke (p = 0.0228 and p = 0.0229, respectively).

To explore potential underlying mechanisms for this outcome, we performed histological analyses of the brains harvested at 14 days post-tMCAo, when endogenous post-stroke neurorepair processes are highly active.^19^

Cortical peri-infarct vessel density was unchanged between ipsilateral and contralateral brain hemispheres in both groups (p > 0.05 for all comparisons vs. IL-1α^fl/fl^; Fig. S6). However, subcortical vessel density was different both between hemispheres (p = 0.0003) and genotypes (p = 0.0152), with significant interaction between hemispheres and genotypes (p = 0.0441). Specifically, peri-infarct subcortical vessel density was enhanced in both IL-1α^fl/fl^ and IL-1α^fl/fl^:Cx3cr1^CreERT2^ mice by 1.6-fold (p = 0.0008 vs. contralateral) and by 1.4-fold (p = 0.0269 vs. contralateral), respectively (Figure 4(c)), which is in line with the ischemic lesion primarily affecting subcortical areas in this model (Figure 2(d)). Furthermore, we observed that IL-1α^fl/fl^:Cx3cr1^CreERT2^ mice had a 4.9-fold decrease in the peri-infarct subcortical vessel density compared to that of IL-1α^fl/fl^ (p = 0.0025; Figure 4(c)). Along with a decreased vessel density at 14 days post-stroke, mice with microglial IL-1α depletion showed a reduced density of reactive astrocytes, measured by the GFAP+ area in the subcortical peri-infarct area (p = 0.0085 vs. IL-1α^fl/fl^ mice; Figure 4(d)).

Overall, microglial IL-1α deletion led to reduced vascular density and astrogliosis, which are known to contribute to post-stroke recovery and could therefore explain the long-term functional impairment amongst IL-1α^fl/fl^:Cx3cr1^CreERT2^ mice, suggesting a possible role of microglial IL-1α in post-stroke neurorepair processes.

On the other hand, both IL-1α^fl/fl^ and IL-1α^fl/fl^:Cx3cr1^CreERT2^ mice showed a similar improvement at 42 days, with no significant differences in the neurological score between genotypes (p = 0.2180). While changes in both functional outcome and neurorepair processes were observed at 14 days, no significant differences associated with microglial IL-1α deletion were shown in either vessel density or astrogliosis at 42 days (p > 0.05 for all measurements vs. IL-1α^fl/fl^; Fig. S7), in line with the neurological score. However, a trend in the source of variation of subcortical vessel density was observed between hemispheres (p = 0.0998) and interaction between brain hemispheres and genotypes (p = 0.0776). Specifically, even though peri-infarct subcortical vessel density was no longer significantly enhanced compared to the contralateral subcortex in either of the genotypes, as seen at 14 days, a trend was observed only amongst IL-1α^fl/fl^ mice by 1.6-fold (p = 0.0532 vs. contralateral).

## Discussion

In this study, we show that IL-1α and IL-1β have differential spatio-temporal expression profiles in the brain after stroke, confirming earlier observations^8^ and suggesting that they might exert distinct non-overlapping roles at different stages of post-stroke inflammation. Here, we show that IL-1α is expressed exclusively by microglial cells in the brain early after stroke onset, followed by a delayed IL-1β expression by a small subset of microglia and mostly by infiltrating neutrophils. Upon stroke onset, microglia are the first resident immune responders, rapidly migrating and becoming activated at the lesion site, exacerbating tissue injury by releasing pro-inflammatory cytokines and cytotoxic factors, but also contributing to tissue repair and remodeling by engulfing cellular debris and invading neutrophils, and releasing anti-inflammatory cytokines and growth factors.^20,21^ With microglia being the major source of brain IL-1α expression and considering its pivotal role in post-stroke neuroinflammation, we aimed to study the specific role of microglial IL-1α in ischemic stroke using our novel microglial-specific IL-1α deficient mouse.

A previous study showed that, whilst the ubiquitous and chronic deletion of both IL-1α and IL-1β significantly reduced ischemic brain damage, the deletion of neither IL-1α nor IL-1β alone had no effects on ischemic brain injury, suggestive of potential compensatory mechanisms.^22^ Ubiquitous KO models, deleting global IL-1α or IL-1β from birth, cannot discern the specific role of IL-1 isoforms of systemic or brain origin, while novel genetic tools currently available, such as conditional and cell-specific KO, provide refined tools to elucidate specific mechanisms in health and disease. Here, using a conditional and cell-specific KO model, we studied for the first time the specific contribution of microglial derived IL-1α in the context of ischemic stroke. In the present study, conditional microglial IL-1α deletion prior to stroke did not influence acute brain damage and cerebral blood flow changes, nor infarct lesion progression as seen by MRI up to 8 days post-stroke in a model of permanent cerebral ischemia. To enhance translatability, we also assessed acute brain damage in two different intraluminal tMCAo models in different research centers, which replicated the lack of effect of microglial IL1-α deletion on infarct size, suggesting that microglial IL-1α does not contribute to acute brain damage, despite its very early expression.

The effect of microglial-derived IL-1α on microglial function in stroke is largely unknown. However, microglial proliferation is known to be the main source of microgliosis after ischemic stroke^23^ and, in this regard, *IL1R1* and *IL1A* have been identified as key genes involved in microglial replenishment upon ablation in adult mice,^24^ while CX3CR1 has been implicated in microglial chemotaxis, microglia-mediated neurotoxicity, and microglial activation.^25^ With all this in mind, we investigated whether Cx3cr1-Cre-mediated microglial IL-1α deletion could affect microglial functions, such as microgliosis and neutrophil engulfment, after both transient and permanent cerebral ischemia. Microglial IL-1α ablation did not modify microglial density nor activation, neutrophil infiltration, endothelial activation, nor IL-1β expression in the brain after stroke. These results suggest that microglial IL-1α may not be involved in microglial functions nor IL-1β expression early after stroke, although future studies should include additional timepoints to elucidate potential microglial IL-1α effects on microglia in the subacute and chronic phases.

Next, we investigated the effect of microglial IL-1α expression on long-term outcome after stroke in the intraluminal model, which allows us to investigate behavioral deficits beyond the acute phase. Surprisingly, despite the early expression of microglial IL-1α, which does not seem to have major effects on acute stroke outcome, its deletion was associated with a worse functional recovery from 14 days and up to 28 days after cerebral ischemia. We also observed that the same microglial IL-1α deficient mice had a reduced peri-infarct vessel density at 14 days post-stroke, suggestive of impaired post-stroke angiogenesis. Peri-infarct vascular remodeling, which can begin as early as 3 days and occurs mostly during the first 2 weeks post-stroke in rodent models, is known to contribute to functional recovery that is associated with restored blood flow.^19,26,27^ These results could therefore explain the behavioral deficits in microglial IL-1α deficient mice and are in line with our previously published work showing that IL-1α treatment enhances angiogenesis both *in vitro*^9^ and *in vivo* alongside improving functional recovery after experimental stroke.^10^ Furthermore, impaired angiogenesis occurred along with reduced astrogliosis as seen by a decreased GFAP expression and slightly lower glial scar thickness. Microglia comprise a highly heterogeneous and plastic cell population, displaying numerous overlapping functional states and participating in a complex crosstalk with different cell types within the neurovascular unit and infiltrating peripheral leukocytes.^20^ In this context, microglia, which respond earlier to injury, can modulate astrocyte activation and functions. Recently, Huang and colleagues proposed that microglia may inhibit the recruitment of neutrophils and secondary occlusions through the release of IL-1RA, which in turn modulates astrocytic CXCL1 expression.^28^ In line with our results, a recent study showed that activated microglia induce reactive astrocytes through the release of IL-1α, TNFα, and C1q; however, this was associated with the activation of neurotoxic reactive astrocytes.^29^ The role of reactive astrocytes in stroke is a subject of debate, with evidence suggesting they can both hinder and promote stroke recovery. In accordance with our results, Williamson and colleagues showed that reactive astrocytes facilitate vascular repair and remodeling, improving motor recovery after stroke.^30^ Although neurorepair processes were observed at 14 days, no significant differences in vessel density or astrogliosis were detected at 42 days, in line with the neurological score. These results suggest that while IL-1α may play a role in early recovery, its influence on chronic repair is less pronounced, which could indicate a window of opportunity to enhance microglial IL-1α-related repair processes in more subacute phases. However, more exhaustive and integrative omics studies are warranted to gain a better understanding of the downstream effects of stroke-induced microglial IL-1α expression.

The present study is not exempt from limitations. Female mice were initially excluded to reduce variability and the total number of animals undergoing procedures. However, future studies should include both sexes as well as relevant stroke comorbidities to enhance translatability. Here, we aimed to investigate the role of IL-1α in acute and subacute stages of ischemic stroke, under conditions of both permanent occlusion and during reperfusion injury after large vessel occlusions. The use of diverse pre-clinical stroke models mimicking the heterogenous clinical setting is critical to comprehensively investigate the pathophysiology of ischemic stroke. However, the use of different models with varying experimental designs can limit the generalizability of our findings, and a cautious and model-dependent interpretation of the data should be done. There is consensus that microglia are long-lived, but they experience turnover albeit at a slower rate compared to peripheral cells. Previous work by Wieghofer et al. using tamoxifen-induced Cx3cr1^CreER^:R26-yfp mice has shown that CX3CR1+ microglia do not undergo significant turnover even several months after tamoxifen induction, while CX3CR1+ monocytes are rapidly replaced.^31^ Microglial turnover is an area of ongoing debate and therefore further studies are warranted to specifically examine microglial turnover and IL-1α expression post-depletion. However, no IL-1α+ microglia were found at 4 h nor 24 h post-stroke upon acute microglial IL-1α+ deletion, indicating that the observed outcomes were not affected by microglial turnover.

Our findings propose that microglial-derived IL-1α does not modify acute stroke outcome, despite its hyperacute expression, yet it may influence long-term cerebrovascular responses modulating functional recovery after experimental stroke. Nonetheless, the exact mechanisms by which early microglial IL-1α modulates neurorepair processes and recovery, as well as the non-redundancy of IL-1α and IL-1β, and the interplay with other cell types are not fully understood. Beyond the early mortality and morbidity due to the acute ischemic brain injury per se, most stroke survivors will develop secondary post-stroke complications, such as cognitive decline, independently of known vascular dementia risk factors. However, the mechanisms linking acute injury to chronic complications are largely unknown.^32^ A recent study suggested that acute IL-1β expression leads to chronic secondary organ dysfunction after stroke through epigenetic changes in myeloid innate immune memory.^33^ It is also known that IL-1α, unlike IL-1β, can translocate into the nucleus, where it exerts transcriptional activity.^34^ Whether the underlying mechanisms of the potential benefits of microglial-IL-1α on post-stroke recovery are driven by early transcriptional modifications leading to long-term microglial phenotypic changes or paracrine effects involving the interplay of cell intermediators participating in neurorepair processes at different stages, remains to be elucidated.

Taken together, this study proposes a critical role for microglial IL-1α in stroke, which should be therefore considered as a potential therapeutic target. Future studies focusing on the specific role of IL-1 family members in stroke are needed to advance the understanding of stroke pathophysiology, which is essential to refine IL-1 targeted stroke interventions.

## Supplemental Material

sj-pdf-1-jcb-10.1177_0271678X251323371 - Supplemental material for Selective deletion of interleukin-1 alpha in microglia does not modify acute outcome but may regulate neurorepair processes after experimental ischemic stroke Supplemental material, sj-pdf-1-jcb-10.1177_0271678X251323371 for Selective deletion of interleukin-1 alpha in microglia does not modify acute outcome but may regulate neurorepair processes after experimental ischemic stroke by Eloïse Lemarchand, Alba Grayston, Raymond Wong, Miyako Rogers, Blake Ouvrier, Benjamin Llewellyn, Freddie Webb, Nikolett Lénárt, Ádám Dénes, David Brough, Stuart M Allan, Gregory J Bix and Emmanuel Pinteaux in Journal of Cerebral Blood Flow & Metabolism
