# Supplementary material for: Selective deletion of interleukin-1 alpha in microglia does not modify acute outcome but may regulate neurorepair processes after experimental ischemic stroke
Source: J Cereb Blood Flow Metab. 2025 Mar 20;45(8):1479–92. doi: 10.1177/0271678X251323371 (PMC11926816; doi:10.1177/0271678X251323371)
Supplement: sj-pdf-1-jcb-10.1177_0271678X251323371 - Supplemental material for Selective deletion of interleukin-1 alpha in microglia does not modify acute outcome but may regulate neurorepair processes after experimental ischemic stroke [file sj-pdf-1-jcb-10.1177_0271678X251323371.pdf]

Supplementary File for

Selective deletion of interleukin-1 alpha in microglia does not modify acute outcome but may regulate neurorepair processes after experimental ischemic stroke.

Running headline: Microglial IL-1α and neurorepair after stroke

Eloïse Lemarchand[1,2,*,†]; Alba Grayston[1,2,*]; Raymond Wong[1,2,*]; Miyako Rogers[1,2]; Blake Ouvrier[3]; Benjamin Llewellyn[1,2]; Freddie Webb[1,2]; Nikolett Lénárt[4]; Ádám Dénes[4]; David Brough[1,2]; Stuart M Allan[1,2]; Gregory J Bix[3]; Emmanuel Pinteaux[1,2].

[*]These authors contributed equally to this work.

[1]Division of Neuroscience, School of Biological Sciences, Faculty of Biology, Medicine and Health (FBMH), The University of Manchester, Manchester, UK.

[2]Geoffrey Jefferson Brain Research Centre, University of Manchester, Northern Care Alliance NHS Foundation Trust, The Manchester Academic Health Science Centre, Manchester, UK.

[3]Department of Neurosurgery, Clinical Neuroscience Research Center, Tulane University School of Medicine, Orleans, LA, USA.

[4]"Momentum" Laboratory of Neuroimmunology, HUN-REN Institute of Experimental Medicine, Budapest, Hungary.

[†]Current affiliation: Normandie University, UNICAEN, INSERM UMR-S U1237, Physiopathology and Imaging of Neurological Disorders, GIP Cyceron, Institute Blood and Brain @ Caen-Normandie, Caen, France.

Correspondence

Dr Emmanuel Pinteaux; FBMH, University of Manchester, AV Hill Building, Manchester M13 9PT, United Kingdom; emmanuel.pinteaux@manchester.ac.uk.

Dr Gregory Bix; Department of Neurosurgery, Clinical Neuroscience Research Center, Tulane University School of Medicine, New Orleans, LA, USA; gbix@tulane.edu.

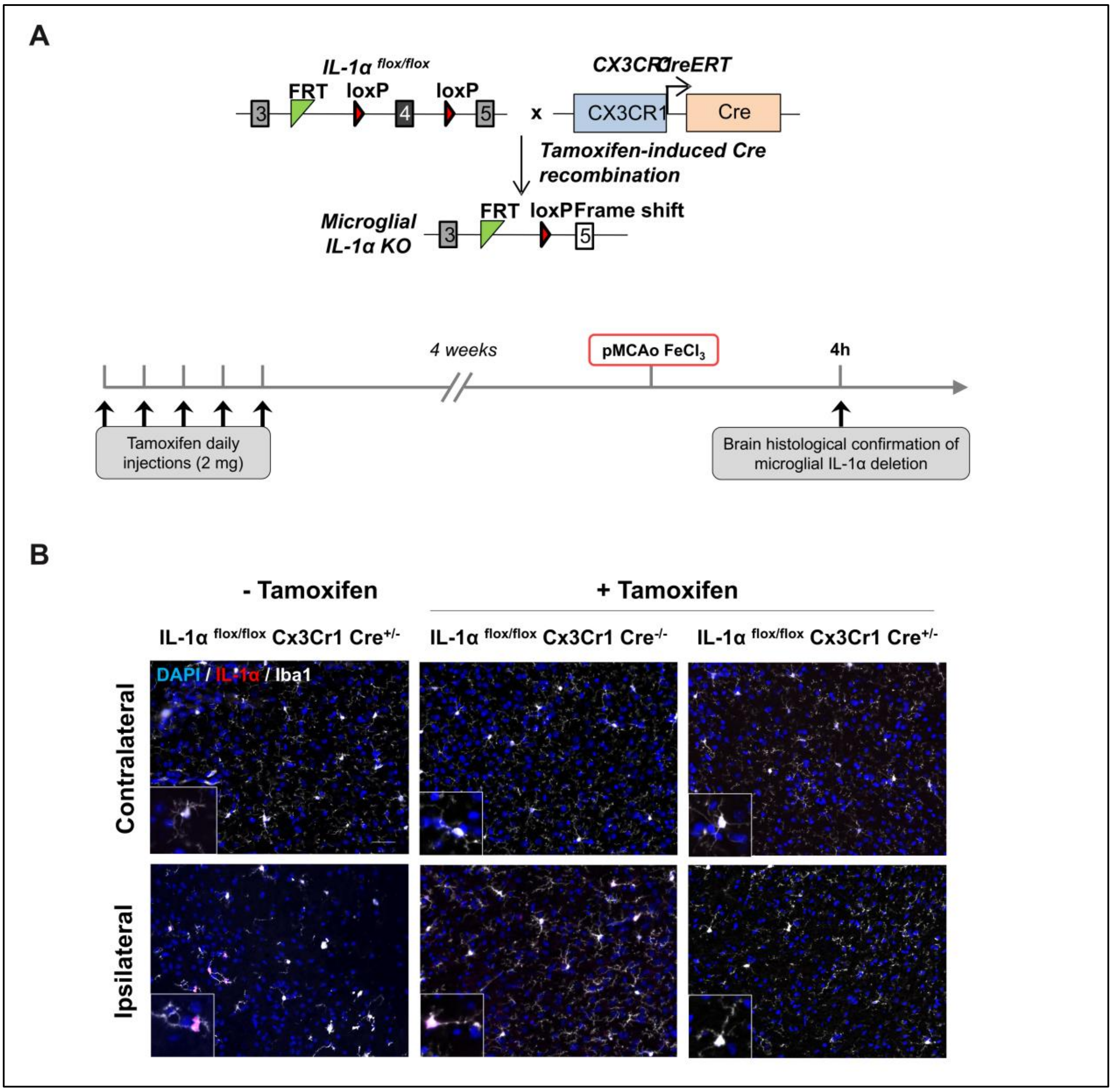

Figure S1. Characterization of a conditional IL-1α mouse mutant crossed with CX3CR1 Cre-ERT2 mice to induce a specific deletion of microglial IL-1α in the brain. (A) Schematic representation of the experimental design. Exon 4 of the IL-1A gene flanked with loxP sites (IL-1α$^{fl/fl}$), is excised upon Cre recombination induced by tamoxifen (in IL-1α$^{fl/fl}$:Cx3cr1-Cre^{ERT2} mice), resulting in the generation of microglia-specific IL-1α KO mice. (B) Representative immunostaining of IL-1α (red), microglia (Iba1, white) and DAPI (blue) in the contralateral, ipsilateral areas at 4 h after pMCAo, showing microglial IL-1α expression abrogation upon tamoxifen administration (Scale bar: 50μm).

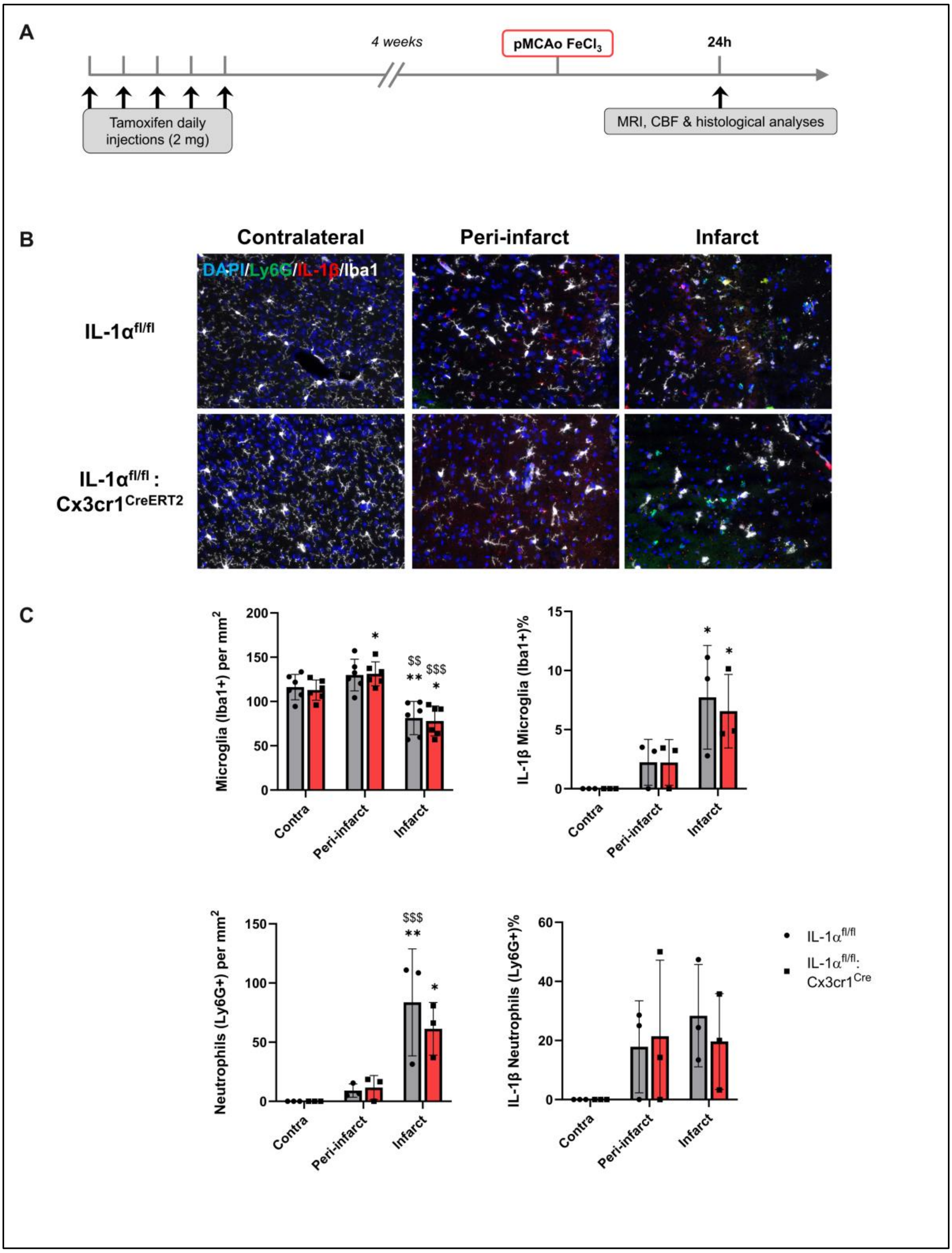

Figure S2. Microglial IL-1α deletion does not influence microglial activation, neutrophil infiltration or IL-1β expression at 24 h after permanent cerebral ischemia. (A) Schematic representation of the experimental design. (B) Representative immunostaining of neutrophils (Ly6G, green), IL-1β (red), microglia (Iba1, white) and DAPI (blue) in the contralateral, peri-infarct and infarct areas at 24 h after pMCAo (Scale bar: 50 µm). (C) Number of microglia (Iba1 positive cells), neutrophils (Ly6G positive cells), percentage of IL-1β positive microglia and IL-1β positive neutrophils

in the contralateral, peri-infarct and infarct areas at 24 h after stroke. (n=6/group, *p<0.05 vs. respective contralateral, **p<0.01 vs. respective contralateral, #p<0.100, $$p<0.01 vs. respective peri-infarct, $$$p<0.001 vs. respective peri-infarct, two-way ANOVA followed by Sidak's post hoc test). Data are shown as mean±SD.

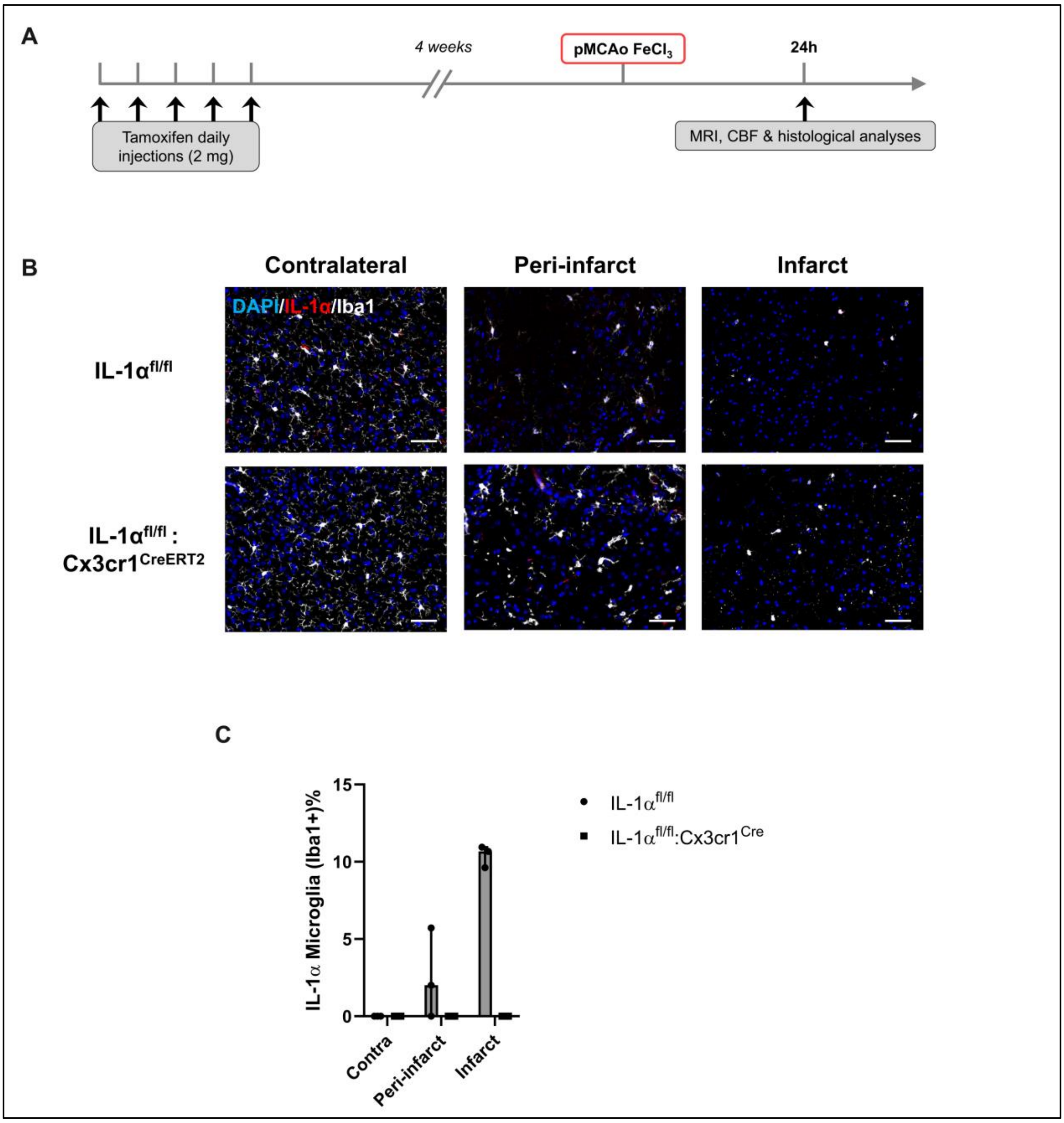

Figure S3. Microglial IL-1α deletion confirmation at 24 h after permanent cerebral ischemia. (A) Schematic representation of the experimental design. (B) Representative immunostaining of IL-1α (red), microglia (Iba1, white) and DAPI (blue) in the contralateral, peri-infarct and infarct areas at 24 h after pMCAo (Scale bar: 50 µm). (C) Percentage of IL-1α positive microglia in the contralateral, peri-infarct and infarct areas at 24 h after stroke. (n=3/group). Data are shown as median (IQR).

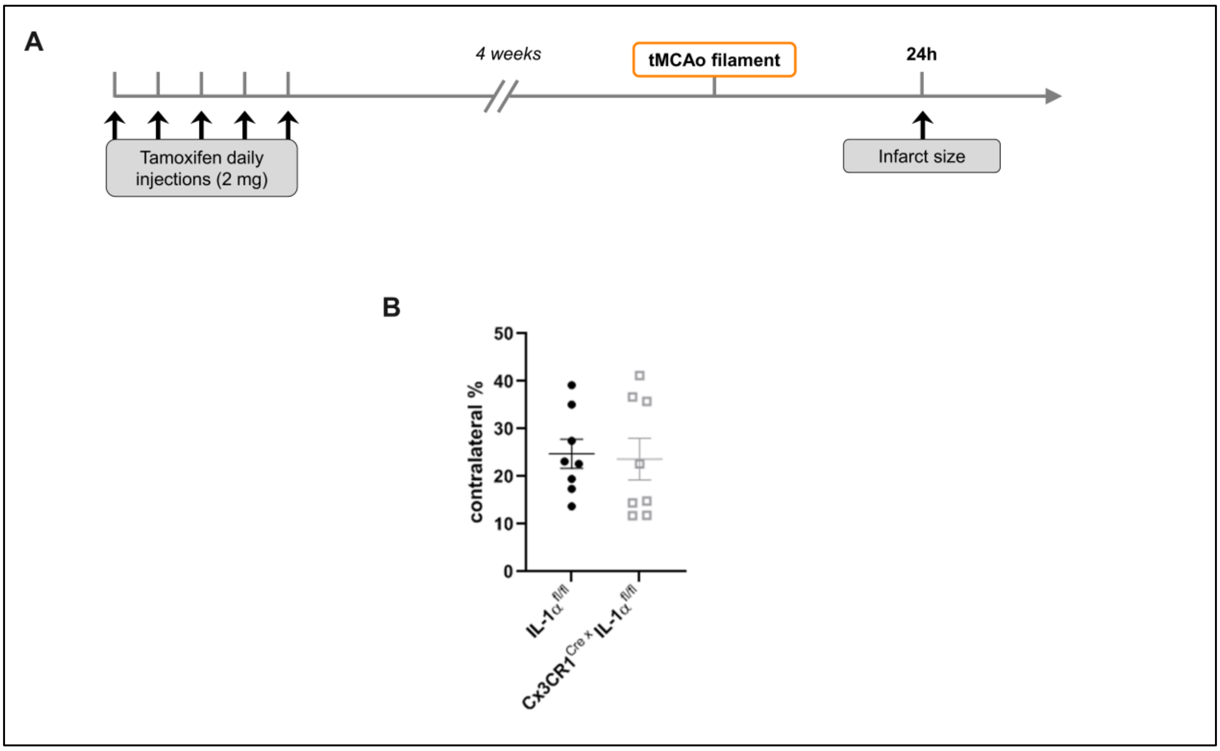

Figure S4. Microglial IL-1α deletion does not influence brain damage at 24 hours after transient cerebral ischemia. (A) Schematic representation of the experimental design to study acute outcome after transient stroke. (B) Infarct percentage at 24 h after tMCAo (45 min occlusion, filament through external carotid artery, ECA) in IL-1$\alpha^{fl/fl}$ and IL-1$\alpha^{fl/fl}$:Cx3cr1-Cre^{ERT2} mice (n=8/group, unpaired t-test). Data are shown as mean±SD.

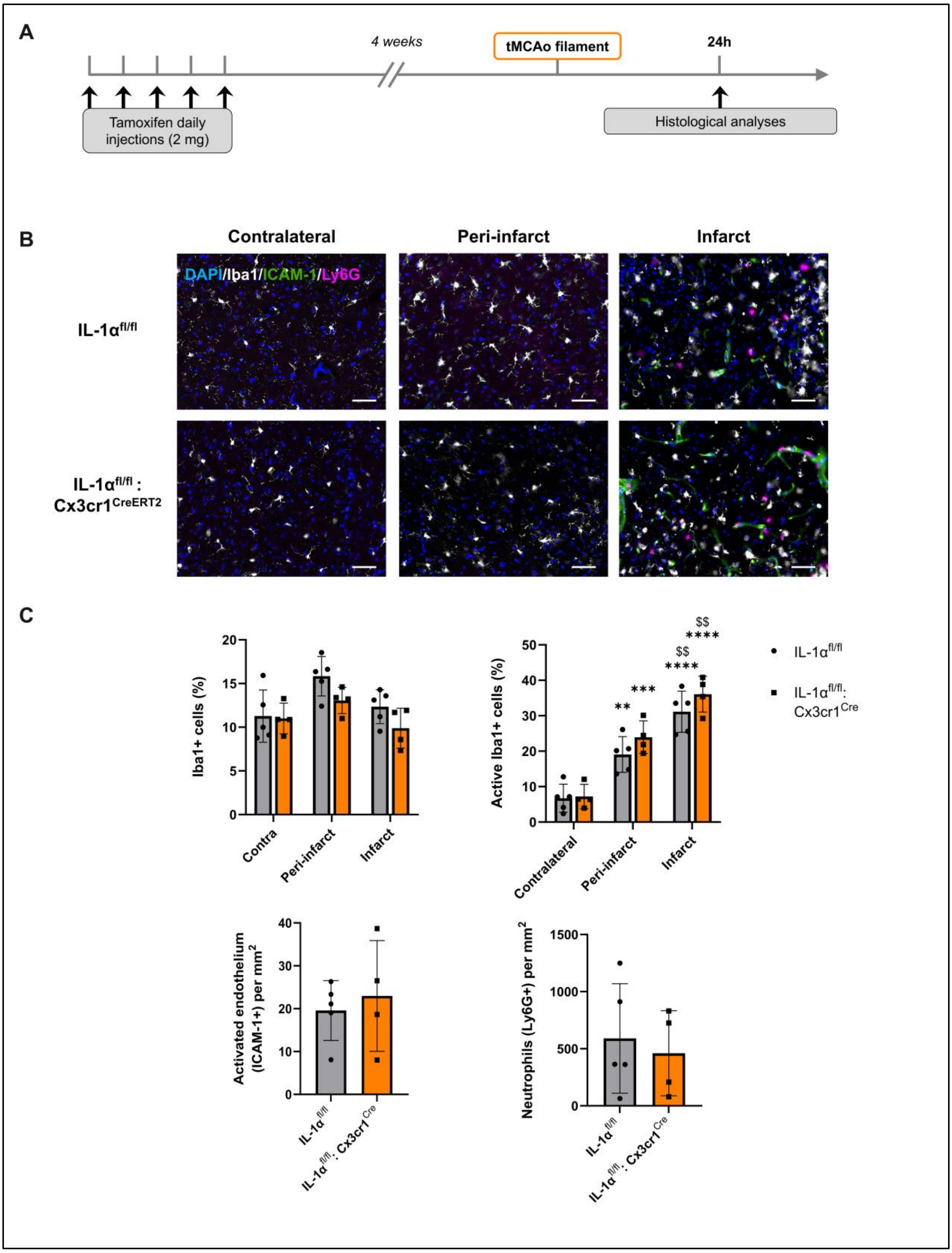

Figure S5. Microglial IL-1α deletion does not influence microglial density and activation, endothelial activation, nor neutrophil infiltration at 24 h after transient cerebral ischemia. (A) Schematic representation of the experimental design. (B) Representative immunostaining of ICAM-1 (activated endothelium, green), neutrophils (Ly6G, purple), microglia (Iba1, white) and DAPI (blue) in the contralateral, peri-infarct and infarct areas at 24 h after tMCAo (Scale bar: 50 µm). (C)

Density of microglia (% of Iba1 positive cells), microglial activation (% of activated Iba1+ cells), endothelial activation (ICAM-1 positive vessels density) and neutrophils (Ly6G positive cells); n=6/group, **p<0.01 vs. respective contralateral, ***p<0.001 vs. respective contralateral, ****p<0.0001 vs. respective contralateral, $$p<0.01 vs. respective peri-infarct, two-way ANOVA followed by Sidak's post hoc test, unpaired t-test. Data are shown as mean±SD.

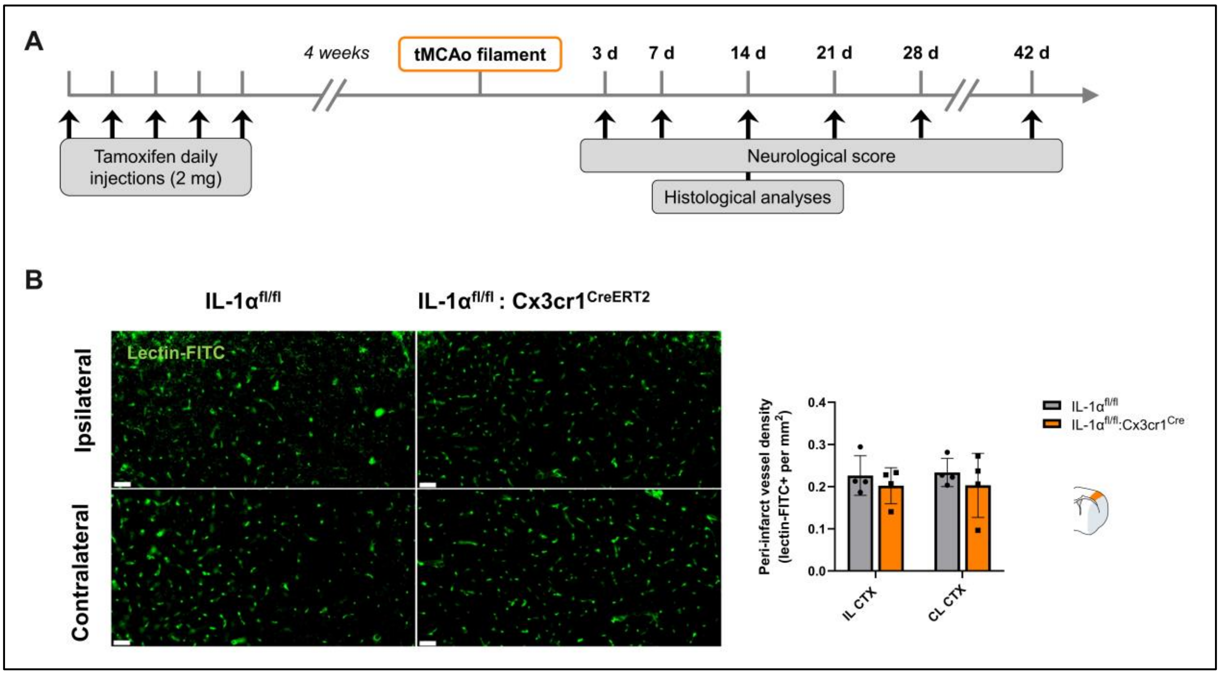

Figure S6. Microglial IL-1α deletion does not affect cortical peri-infarct vessel density at 14 days post-stroke. (A) Schematic representation of the experimental design. (B) Representative immunostaining of vascular density (lectin-FITC+) in the ipsilateral peri-infarct and corresponding contralateral cortical brain regions in IL-1α$^{fl/fl}$ and IL-1α$^{fl/fl}$:Cx3cr1^{ERT2} mice, and quantification (Scale bar: 50 µm, n=4/group, two-way ANOVA followed by Sidak's post hoc test). Data are shown as mean±SD.

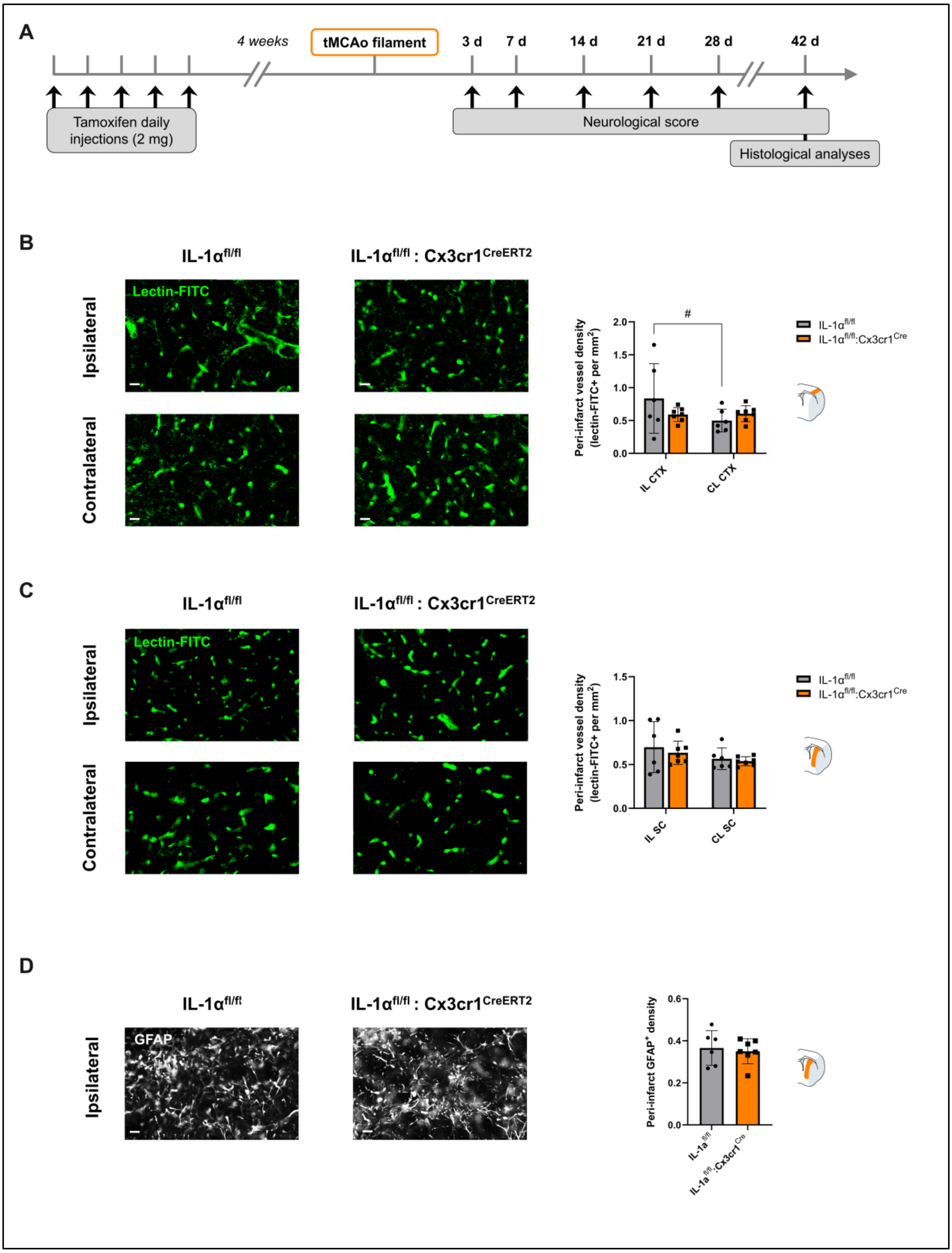

Figure S7. Microglial IL-1α deletion does not affect peri-infarct vessel density nor astrogliosis at 42 days post-stroke. (A) Schematic representation of the experimental design. (B) Representative immunostaining of vascular density (lectin-FITC+) in the ipsilateral peri-infarct and corresponding contralateral cortical brain regions in IL-1α$^{fl/fl}$ and IL-1α$^{fl/fl}$:Cx3cr1^{ERT2} mice, and quantification (Scale bar: 20 µm, n=6/group, #p<0.100, two-way ANOVA followed by Sidak's post

hoc test). (C) Representative immunostaining of vascular density (lectin-FITC+) in the ipsilateral peri-infarct and corresponding contralateral subcortical brain regions in IL-1α$^{fl/fl}$ and IL-1α$^{fl/fl}$:Cx3cr1^{ERT2} mice, and quantification (Scale bar: 20 µm, n=6/group, two-way ANOVA followed by Sidak's post hoc test). (D) Representative immunostaining of the glial scar (GFAP+) in the ipsilateral peri-infarct subcortical brain region in IL-1α$^{fl/fl}$ and IL-1α$^{fl/fl}$:Cx3cr1-Cre^{ERT2} mice, and quantification of the GFAP+ density (Scale bar: 20 µm, n=6-7/group, unpaired t-test, **p<0.01).
